# Dual-defect-modified graphitic carbon nitride with boosted photocatalytic activity under visible light

**DOI:** 10.1038/s41598-019-49949-6

**Published:** 2019-10-16

**Authors:** Hideyuki Katsumata, Fumiya Higashi, Yuya Kobayashi, Ikki Tateishi, Mai Furukawa, Satoshi Kaneco

**Affiliations:** 1 Department of Chemistry for Materials, Graduate School of Engineering, Mie University, Tsu, Mie 514-8507 Japan; 2 Mie Global Environment Center for Education & Research, Mie University, Tsu, Mie 514-8507 Japan

**Keywords:** Photocatalysis, Pollution remediation

## Abstract

The development of photocatalysts that efficiently degrade organic pollutants is an important environmental-remediation objective. To that end, we report a strategy for the ready fabrication of oxygen-doped graphitic carbon nitride (CN) with engendered nitrogen deficiencies. The addition of KOH and oxalic acid during the thermal condensation of urea led to a material that exhibits a significantly higher pseudo-first-order rate constant for the degradation of bisphenol A (BPA) (0.0225 min^−1^) compared to that of CN (0.00222 min^−1^). The enhanced photocatalytic activity for the degradation of BPA exhibited by the dual-defect-modified CN (Bt-OA-CN) is ascribable to a considerable red-shift in its light absorption compared to that of CN, as well as its modulated energy band structure and more-efficient charge separation. Furthermore, we confirmed that the *in-situ*-formed cyano groups in the Bt-OA-CN photocatalyst act as strong electron-withdrawing groups that efficiently separate and transfer photo-generated charge carriers to the surface of the photocatalyst. This study provides novel insight into the *in-situ* dual-defect strategy for g-C_3_N_4_, which is extendable to the modification of other photocatalysts; it also introduces Bt-OA-CN as a potential highly efficient visible-light-responsive photocatalyst for use in environmental-remediation applications.

## Introduction

Photocatalytic semiconductor technology is considered to be useful and promising for the remediation of environmental organic contaminants, because it can effectively decompose pollutants into inorganic compounds, such as CO_2_ and H_2_O. Therefore, discovering highly active photocatalysts continues to be a worldwide effort. Recently, graphitic carbon nitride (g-C_3_N_4_), which is a π-conjugated polymer semiconductor, has captured the attentions of catalysis researchers, due to its unique characteristics that include its high chemical and thermal stabilities, preferable electronic band alignment, lack of toxicity, cost effectiveness, ease of preparation, and visible-light responsiveness^1–8^. It has also stimulated numerous studies on H_2_ evolution through water splitting^1,4,9–11^, organic-pollutant degradation^12,13^, artificial photosynthesis^6,14,15^, and CO_2_ reduction^16,17^. However, g-C_3_N_4_ exhibits poor photocatalytic performance because of its limited ability to absorb visible light, its high rate of hole-electron pair recombination, low specific surface area, and few reactive sites^18–20^. Many strategies have been used in attempts to solve the above-mentioned disadvantages and to improve the photocatalytic activity of g-C_3_N_4_, including morphological modifications^21–25^, elemental doping^26–31^, modifications with non-metallic and metallic materials^32–35^, surface chemical modifications^10,36–38^, and combinations with other photocatalysts to fabricate hybrid composites^12,16,19,39–46^.

The formation of nitrogen defects in the g-C_3_N_4_ framework is an effective way of reducing the charge-transfer barrier and improving its photocatalytic performance under visible light^46^. As a result, the defect-control methodology has been intensively studied with the aim of increasing the visible-light absorption of g-C_3_N_4_ and engender it with superior activity^46–50^. Defects are easily introduced into g-C_3_N_4_ by modifying its synthesis conditions, which is ascribable to its metal-free polymer properties and heterogeneous atomic environment; i.e., the various bonding states of its two- and three-coordinated nitrogen atoms. In particular, the formation of nitrogen defects has recently been demonstrated to significantly enhance the photocatalytic activity of g-C_3_N_4_ under visible light^47^. Nitrogen defects in g-C_3_N_4_ can create mid-gap bands, serve as reactive sites for the excitation of charge carriers, and expand the optical responses of photocatalyst materials. Furthermore, nitrogen defects supply trapping sites for photo-induced charge carriers, preventing their recombination and promoting the overall quantum efficiency^51–53^. Many strategies for generating nitrogen defects in g-C_3_N_4_ have been investigated to date, including polymerization-temperature control^47,53^, hydrothermal treatment^51^, and hydrogen reduction^46,52^. KOH treatment of urea, as a precursor of g-C_3_N_4_, promotes alkali-assisted thermal condensation and is also an effective method for producing nitrogen defects. This method was used to demonstrate that the band alignment of g-C_3_N_4_ can be controlled by the formation of cyano groups and surface nitrogen deficiencies in the g-C_3_N_4_ framework, which are preferable for collecting incident visible light and effectively separating photo-generated hole-electron pairs^54^.

On the other hand, non-metal doping (B, C, O, F, P, and S) has been reported to play a significant role in enhancing solar-light absorption by g-C_3_N_4_ and its photocatalytic activity^55^. In particular, O-doping accelerates the photocatalytic performance of g-C_3_N_4_ due to improvements in its electronic band structure and charge-carrier usage^56–58^. In addition, O-doped g-C_3_N_4_ is relatively easy to fabricate by treating melamine (as a precursor) with H_2_O_2_, or by treating g-C_3_N_4_ with H_2_O_2_ under an oxygen atmosphere^59–61^. Other O-doping methods that use thermal oxidation^62^ and hydrothermal treatment have also been established^63–65^. Recently, Qiu *et al*.^66^ developed a simple, low-cost and green method for the synthesis of O-doped porous g-C_3_N_4_ by condensing oxalic acid with urea; this material displayed a significantly enhanced ability to efficiently degrade contaminants due to its high specific surface area, efficient charge separation, and its intense visible-light-absorption properties (≈700 nm). However, to the best of our knowledge, there are very few reports on dual modifications involving oxygen doping and nitrogen defects in a g-C_3_N_4_ framework^67,68^.

In this work, we introduce a facile method for the synthesis of dual-modified O-doped g-C_3_N_4_ containing nitrogen defects through the thermal polymerization of urea treated with KOH and oxalic acid. The dual-defect-modified g-C_3_N_4_ (Bt-OA-CN) exhibits an intense red-shift in light absorption as well as a modulated energy band structure and more-effective separation of charge carriers compared to g-C_3_N_4_. Because of its improved visible-light absorption and efficient charge separation, the Bt-OA-CN photocatalyst exhibits higher photocatalytic activity for the degradation of bisphenol A (BPA) compared to pristine g-C_3_N_4_ (CN), N-defect CN (Bt-CN), and O-doped CN (OA-CN) when irradiated with visible light. The excellent photocatalytic performance of Bt-OA-CN is ascribable to synergism between the oxygen dopants and the nitrogen defects, as well as the introduction of cyano moieties, which serve as strong electron-withdrawing groups that efficiently separate and transfer photo-induced charge carriers to the surface of the photocatalyst.

## Results and Discussion

### Characterizing the photocatalysts

**Figure 1:**
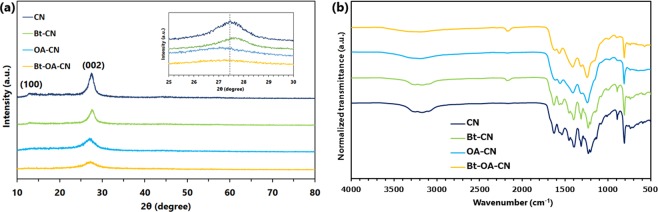
(**a**) XRD patterns and (**b**) FTIR spectra of CN, Bt-CN, OA-CN, and Bt-OA-CN.

As shown in Fig. 1, all samples exhibit very similar powder X-ray diffraction (XRD) patterns and Fourier-transform infrared (FTIR) spectra, indicating that the treated samples (Bt-, OA- and Bt-OA-CNs) retain the general CN structure. The XRD pattern of CN exhibits two characteristic peaks at 13.0° and 27.5°, assigned to the (100) and (002) planes, which correspond to in-plane packing and the characteristic interlayer stacking of aromatic systems, respectively^69^. Fig. 1a clearly shows that the (002) peak shifts to higher angle in Bt-CN, but to lower angles in OA- and Bt-OA-CNs compared to CN, suggesting a decrease and increases in interlayer distance in Bt-CN, and OA- and Bt-OA-CNs, respectively^70^. In addition, both peaks in the spectra of the treated samples were broader and weaker than those in the spectrum of CN, which is ascribable to decreases in the ordered structure within the g-C_3_N_4_ framework.

The normalized FTIR spectrum of CN (Fig. 1b) exhibits multiple peaks in the 1200–1600 cm^−1^ range that are attributable to the typical C-N stretching modes of C-N heterocycles^71^. The sharp peak at 810 cm^−1^ and the broad ones in the 3000–3400 cm^−1^ range are due to the breathing mode of the heptazine units and N-H stretching modes, respectively^72^. The spectra of the samples prepared with KOH (Bt-CN and Bt-OA-CN) exhibit additional peaks at 2177 cm^−1^ that correspond to the asymmetric stretching mode of the cyano group^54^. Clearly, the addition of KOH during the preparation of g-C_3_N_4_ promotes the formation of cyano groups in its framework. On the other hand, the peaks at 1238 cm^−1^, which corresponds to the C-N stretching vibration, were clearly broader in the spectra of the oxalic-acid-added samples (OA-CN and Bt-OA-CN), which indicates that some of the C-N bonds were replaced with C-O bonds^66^. Furthermore, markedly lower normalized intensities of the broad peaks between 3000 and 3400 cm^−1^ were observed in the spectra of the OA-CN and Bt-OA-CN photocatalysts. These results indicate that the use of oxalic acid decreases the concentration of N-H groups, suggestive of smaller planar layers.

**Figure 2:**
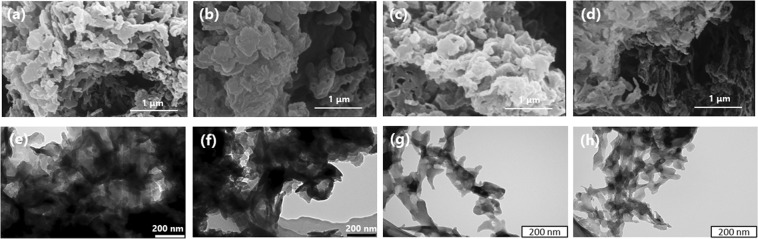
(**a**–**d**) SEM and (**e**–**h**) TEM images of (**a**,**e**) CN, (**b**,**f**) Bt-CN, (**c**,**g**) OA-CN, and (**d**,**h**) Bt-OA-CN.

The morphologies of CN, Bt-CN, OA-CN, and Bt-OA-CN were observed by scanning electron microscopy (SEM) and transmission electron microscopy (TEM), the results of which are shown in Fig. 2. The SEM and TEM images reveal that all samples possess sheet-like structures. Therefore, the additions of KOH and oxalic acid preserved the characteristic sheet structure and thin layers of g-C_3_N_4_. On the other hand, the OA-CN and Bt-OA-CN samples were fragmented, suggesting that oxalic acid reacts with urea as the precursor during thermal polymerization to cause fragmentation of the g-C_3_N_4_ framework^73^. These results are in good agreement with the XRD and FTIR results. To further understand the specific surface areas and pore size distributions of these catalyst, the samples were subjected to N_2_ adsorption–desorption experiments. All samples exhibited typical type-IV isotherms with hysteresis loops, indicating the presence of mesopores (Fig. S1a). The Brunauer–Emmett–Teller (BET) specific surface areas of CN and OA-CN were both 54.6 m^2^/g, whereas those of Bt-CN and Bt-OA-CN were smaller, at 34.7 and 32.4 m^2^/g, respectively (Table 1). CN exhibits three peaks at about 3, 30, and 45 nm in its Barrett–Joyner–Halenda (BJH) pore size distribution profile, while the profiles of the treated catalysts were different (Fig. S1b). Therefore, we conclude that the addition of KOH modifies the BET surface area and BJH pore distribution of CN, while the addition of oxalic acid only affects its BJH pore distribution.

**Table 1:** BET specific surface areas, total pore volumes, and average pore diameters of CN, Bt-CN, OA-CN, and Bt-OA-CN.

Photocatalyst	BET specific surface area (m^2^ g^−1^)	Total pore volume (cm^3^ g^−1^)	Average pore diameter (nm)
CN	54.6	0.39	28.5
Bt-CN	34.7	0.22	25.9
OA-CN	54.6	0.26	18.9
Bt-OA-CN	32.4	0.22	26.7

**Figure 3:**
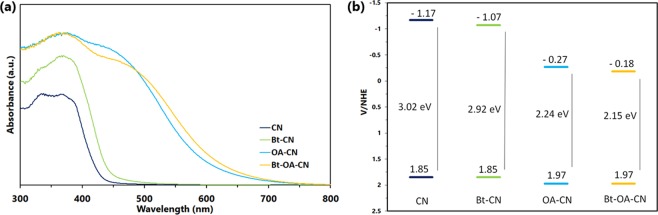
(**a**) UV-vis DRS spectra and (**b**) band structure alignments of CN, Bt-CN, OA-CN and Bt-OA-CN.

The light absorption properties of the resultant samples were explored by UV–vis diffuse reflectance spectroscopy (DRS) (Fig. 3a). CN exhibits an intrinsic absorption edge at around 460 nm, with a calculated band gap of 3.02 eV as determined by the Kubelka–Munk method (Fig. S2a). The modified catalysts exhibit expanded absorption wavelength regions compared to that of CN. In particular, Bt-OA-CN is able to absorb above 740 nm. The corresponding bandgaps of Bt-CN, OA-CN, and Bt-OA-CN were estimated to be 2.92, 2.24, and 2.15 eV, respectively. Therefore, the addition of KOH and oxalic acid during the thermal condensation of urea drastically modified the optical features and light-harvesting capability of g-C_3_N_4_. To explain the narrower bandgaps of the treated CNs, the samples were subjected to valence-band (VB) X-ray photoelectron spectroscopy (XPS) (Fig. S2b). The VB positions of both CN and Bt-CN were found to be similar (about 1.85 eV) whereas those of OA-CN and Bt-OA-CN are downshifted to 1.97 eV, which is ascribable to the higher O2p orbital energy compared to that of the N2p orbital through the introduction of O atoms (*vide infra*)^74^. On the other hand, the N-defects described below have little effect on the VB position of g-C_3_N_4_. The DRS and VB-XPS results reveal that the narrower bandgaps of the modified samples originate from lower conduction-band (CB) energies, compared with that of g-C_3_N_4_; the schematic band structures are shown in Fig. 3b.

**Figure 4:**
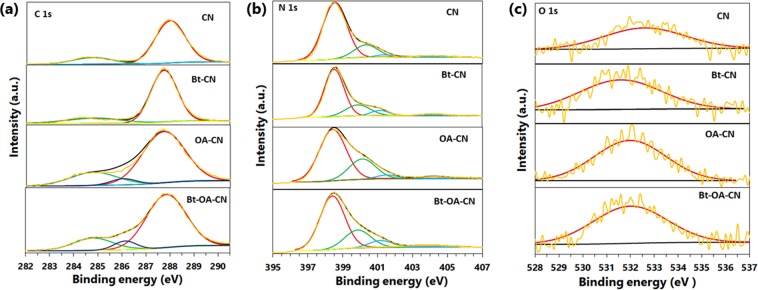
High resolution (**a**) C1s, (**b**) N1s, and (**c**) O1s XPS spectra of CN, Bt-CN, OA-CN, and Bt-OA-CN.

In order to further understand the effects of adding KOH and oxalic acid on the surface chemical composition and elemental chemical state of g-C_3_N_4_, the samples were subjected to XPS. The atomic ratios in the CN, Bt-CN, OA-CN, and Bt-OA-CN samples were determined from their XPS survey spectra (Fig. S3). The N/C atomic ratio of the CN sample was found to be 1.30, which is very close to the theoretical value (Table 2). However, the XPS results showed a significantly lower N/C ratio (1.23) on the surface of g-C_3_N_4_ prepared by the addition of KOH and oxalic acid, which suggests that surface N defects have been introduced. Furthermore, clear O1s peaks were found in the spectra of OA- and Bt-OA-CN, indicating that N atoms in the g-C_3_N_4_ framework had been substituted with O atoms. To further confirm that the addition of KOH and oxalic acid introduces O-dopants and N-defects, high-resolution XPS spectra of CN, Bt-CN, OA-CN and Bt-OA-CN were acquired and divided into their chemical states (Fig. 4). The C1s XPS spectra of CN and Bt-CN are composed of two peaks at 284.7 and 288.0 eV that correspond to graphitic carbon and N-C=N coordination, respectively (Fig. 4a)^75,76^. We observed that the C1s spectra of OA- and Bt-OA-CN exhibit a new component located at 286.0 eV, which is attributed to C-O bonding^76^. As seen in Table S1, the N-C = N ratios were remarkably lower, while the C-O ratios were remarkably higher in the OA- and Bt-OA-CN samples, compared with those of CN, reflecting the replacement of the N atoms in the g-C_3_N_4_ framework with O atoms in the OA- and Bt-OA-CN samples. Therefore, we conclude that the addition of oxalic acid during the thermal condensation of g-C_3_N_4_ leads to O-doping in the g-C_3_N_4_ framework. In addition, the N1s spectrum of CN was fitted by four peaks at 398.5, 400.3, 401.2, and 404.0 eV that correspond to triazine rings (C-N=C), tertiary nitrogen (N–(C)_3_), amino functions (N–H), and π-excitation, respectively^75^. The spectra of Bt- and Bt-OA-CN exhibit tertiary-nitrogen peaks that are located at slightly lower binding energies than that of CN, which is ascribable to the formation of cyano groups (Table S2 and Fig. 4b)^77^. These results are in good accordance with the FTIR observations (Fig. 1b). Furthermore, the C-N=C ratios of Bt-, OA- and Bt-OA-CN were lower compared to that of CN (from 0.77 to 0.66), which provides strong evidence that N-defects and O-dopants were formed at the secondary nitrogen (C-N=C) atoms of the g-C_3_N_4_-framework surface by the addition of KOH and oxalic acid during thermal polymerization. The O1s XPS spectra of CN and Bt-CN (Fig. 4c) each exhibit one component, at 532.6 and 531.6 eV, respectively, which are attributed to water and adsorbed CO_2_^78^. On the other hand, the O1s spectra of OA- and Bt-OA-CN exhibit peaks at 532.0 eV that correspond to C-O-C species^78^. The O1s spectra clearly support the introduction of C-O bonds in the g-C_3_N_4_ framework.

**Table 2:** XPS-determined surface atomic ratios and N/Cs of CN, Bt-CN, OA-CN, and Bt-OA-CN.

Photocatalyst	Atomic ratio (%)			
	C	N	O	N/C
CN	43.0	56.0	1.0	1.30
Bt-CN	44.1	54.1	1.8	1.23
OA-CN	43.0	54.3	2.8	1.26
Bt-OA-CN	43.5	53.4	3.1	1.23

**Figure 5:**
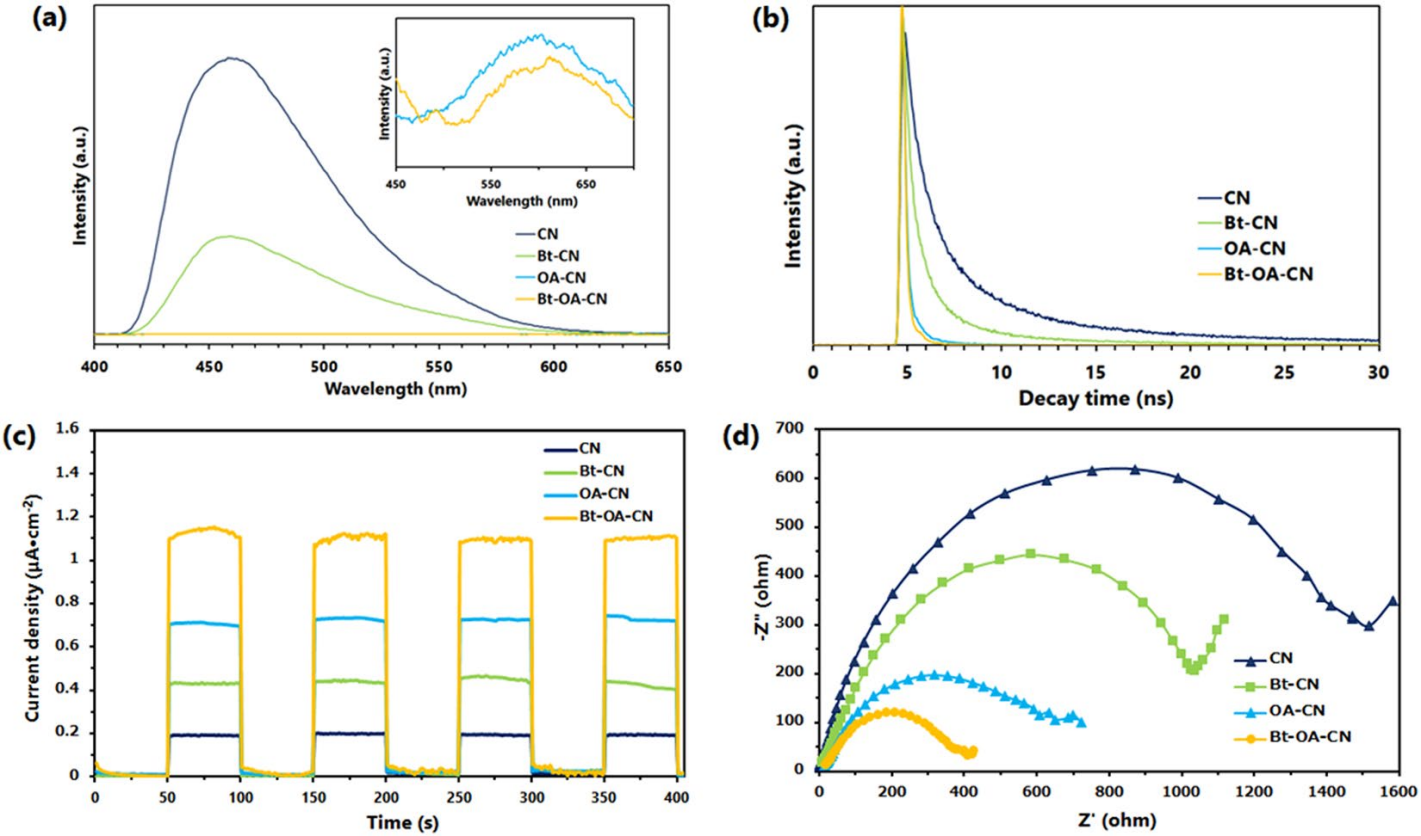
(**a**) PL spectra (inset: enlarged PL spectra of OA-CN and Bt-OA-CN), (**b**) TRPL spectra, (**c**) transient photocurrent responses, and (**d**) EIS Nyquist plots of CN, Bt-CN, OA-CN, and Bt-OA-CN.

In addition to their effects on the bandgaps and the chemical structures, the N-defects and O-dopants in g-C_3_N_4_ are also presumed to affect the photogenerated charge separation, which was examined by photoluminescence (PL) and time-resolved fluorescence-decay (TRPL) experiments. The PL spectra of CN and the modified CNs are shown in Fig. 5a. The PL peaks are related to the radiative recombination of photo-excited electron-hole pairs^39^. Compared to CN, the Bt-CN, OA-CN, and Bt-OA-CN samples exhibited remarkable fluorescence-quenching behaviour (particularly Bt-OA-CN), which suggests that photogenerated electron delocalization at the N-defect and O-replacement sites, as well as the cyano groups, lower the surface traps for charge-carrier recombination in the modified photocatalysts. The TRPL results are shown in Fig. 5b. All samples exhibit decay curves that are well fitted to the following equation^79^:

1 $${I}_{(t)}={A}_{1}\exp (-\frac{t}{{\tau }_{1}})+{A}_{2}\exp (-\frac{t}{{\tau }_{2}})+{A}_{3}\exp (-\frac{t}{{\tau }_{3}}),$$

where A_1_, A_2_, and A_3_ denote pre-exponential factors, and τ_1_, τ_2_, and τ_3_ are fluorescence-decay lifetimes. The average lifetime (<τ>) was calculated using the following equation^79^:

2 $$ < \tau  > =\frac{{A}_{1}{\tau }_{1}^{2}+{A}_{2}{\tau }_{2}^{2}+{A}_{3}{\tau }_{3}^{2}}{{A}_{1}{\tau }_{1}+{A}_{2}{\tau }_{2}+{A}_{3}{\tau }_{3}}.$$

The calculated data are shown in Table S3. The average decay lifetime of the Bt-OA-CN photocatalyst was determined to be 0.208 ns, which is 35-fold shorter than that of CN (7.85 ns). The observed suppression of the radiative recombination and shortened PL lifetime (Fig. 5a and b) are attributed to the presence of the band-tails-involved rapid recombination processes of electron-hole pairs in g-C_3_N_4_ framework with the defects sites^47,80,81^. The band tails act as shallow trap states of charge carriers, and an increased band tails can enhance the rapid PL processes. The dual-defects in g-C_3_N_4_ framework suppress the recombination of photogenerated charge carriers due to the band-to-tail charge transfer processes induced by the increased band tails. These are favorable to the photocatalytic reactions. The increased band tails resulted from N-defects and O-doping cause the suppression of radiative electron-hole recombination by introducing abundant shallow charge trapping states.

The (photo)electrochemical properties of CN and the modified CNs were measured by electrochemical impedance spectroscopy (EIS) and their transient photocurrent responses. As shown in Fig. 5c, the modified CNs, particularly Bt-OA-CN, display significantly improved photocurrents with respect to CN, implying enhanced charge separation. The EIS Nyquist plots of the modified CNs, particularly Bt-OA-CN, exhibit smaller radii compared to that of CN (Fig. 5d), indicating that structural modification results in improved resistance for charge transfer, leading to more efficient separation, transfer, and migration of charge carriers. These results are consistent with those from PL and TRPL studies. Therefore, dual defects, such as N-deficiencies and O-dopants, can promote charge transfer in the g-C_3_N_4_ framework, which enhances the photocatalytic activity.

### Photocatalytic activities of the photocatalysts

**Figure 6:**
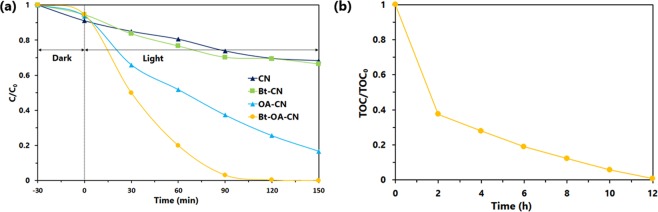
(**a**) Photocatalytic activities of CN, Bt-CN, OA-CN, and Bt-OA-CN toward the degradation of BPA, and (**b**) relative TOC content from BPA over Bt-OA-CN under visible light irradiation as a function of time.

To evaluate the photocatalytic activities of CN and the modified CNs, BPA was degraded under visible light irradiation in activity experiments. The effects of the amounts of KOH and oxalic acid during thermal polymerization on photocatalytic activity were investigated, the results of which are shown in Fig. S4, which clearly reveals that the optimum conditions for the thermal polymerization of g-C_3_N_4_ include the addition of 0.1 g of KOH and 5.0 g of oxalic acid to 15 g of the urea precursor. Figure 6a shows the photocatalytic degradation of BPA as a function of irradiation time over each photocatalyst. As shown in Fig. 6a, each modified g-C_3_N_4_ photocatalyst exhibits higher photocatalytic activity than that of CN. CN shows the lowest photocatalytic activity, with 32% of the BPA degraded after 150 min of irradiation with visible light. Among the modified g-C_3_N_4_ photocatalysts, Bt-OA-CN exhibited the highest photocatalytic activity for the degradation of BPA; BPA was completely degraded within 150 min over Bt-OA-CN. The pseudo-first-order reaction rate constant for the degradation of BPA under visible light over Bt-OA-CN was 0.025 min^−1^, which is 11.4 times greater than that of CN (0.0022 min^−1^).

The wavelength dependence of the photocatalytic activity for the degradation of BPA over Bt-OA-CN was examined using different wavelength band-pass filters (Fig. S5). Degradation efficiency was observed to decrease with increasing wavelength, although BPA degraded over 6 h using Bt-OA-CN even when irradiated with 600-nm light; on the other hand, BPA was not degraded by CN when irradiated at 600 nm. These results suggest that we successfully expanded the incident wavelength range of CN for the degradation of BPA to more useful values through the formation of Bt-OA-CN. BPA solution-mineralization progress over the Bt-OA-CN photocatalyst was monitored by analysing the total organic carbon content (TOC), which was observed to rapidly decrease with increasing irradiation time; the remaining TOC content of the BPA solution was not detected after 12 h of irradiation with visible light (Fig. 6b), reflecting the fact that BPA was completely mineralized into CO_2_ and H_2_O. The TOC results indicate that the use of Bt-OA-CN is effective for not only for removal, but also for the mineralization of BPA in water samples.

In order to examine the photocatalytic stability of the Bt-OA-CN sample under visible light, the same powder sample was used five times after washing with water prior to each run. As seen in Fig. S6, the photocatalytic activity gradually decreased to 83% after five-cycles of use, which is ascribable to the adsorption and accumulation of insoluble intermediate compounds from the degradation of BPA onto the surface of the Bt-OA-CN photocatalyst and/or the loss of photocatalytic particles during stability testing. However, the observed decrease in degradation efficiency is considered to be within tolerance levels; therefore, we conclude that the Bt-OA-CN photocatalyst is highly stable under visible-light irradiation and is expected to be effective for practical applications.

### Photocatalysis mechanism

To investigate the photocatalysis mechanism associated with the Bt-OA-CN catalyst, trapping experiments were conducted in order to determine the predominant photogenerated active species during the degradation of BPA. To capture •OH, h^+^, and O_2_^•−^, *t*-butyl alcohol (TBA), ammonium oxalate (AO), and benzoquinone (BQ), which act as scavengers, were added to the photocatalysis mixture, respectively. As shown in Fig. S7, the degradation of BPA was only weakly inhibited by the addition of TBA, which suggests that •OH is not main active species in this system. On the other hand, the photocatalytic degradation of BPA was clearly lower in the presence of AO and BQ. It is therefore very likely that h^+^ and O_2_^•−^ are the dominant reactive species involved in the photocatalytic degradation of BPA under visible-light irradiation catalysed by Bt-OA-CN.

The band structure also provides important photocatalysis-mechanism clues. Figure 3b reveals that the CB potential for Bt-OA-CN is −0.18 eV, which is higher than the O_2_/O_2_^•−^ redox potential (−0.046 eV vs. NHE)^66^, whereas the VB potential (+1.97 eV) is less positive than that for •OH/H_2_O (+2.27 eV vs. NHE) and •OH/OH^−^ (+1.99 eV vs. NHE)^66^. Therefore, the electrons on the Bt-OA-CN surface are able to react with O_2_ to generate O_2_^•−^, while the holes in the VB are unable to oxidize adsorbed OH^−^ and H_2_O on the catalyst to form •OH directly in the Bt-OA-CN photocatalysis system, which is not inconsistent with the scavenging results described above.

**Figure 7:**
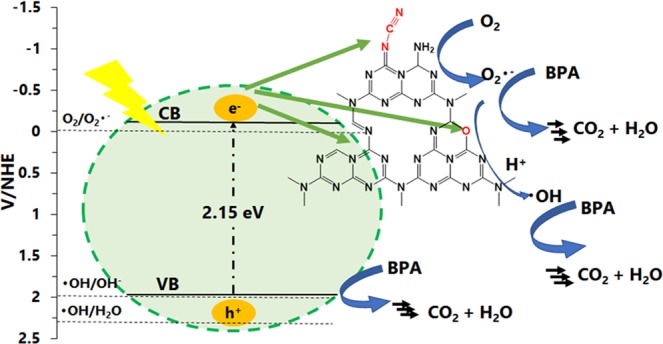
Proposed mechanism for the photocatalytic behaviour of Bt-OA-CN under visible light irradiation.

Based on the above discussion, we propose the enhanced photocatalytic mechanism of the Bt-OA-CN catalyst toward the degradation of BPA shown in Fig. 7. The introduction of O atoms into the heptazine units red-shifts their light-absorption range to over 700 nm, inhibits the recombination of hole-electron pairs, and modifies the positions of the VB and CB; the VB edge moves downwards from 1.85 eV to 1.97 eV, which contributes to the increased oxidizing power of this material for the oxidative degradation of BPA. The N-deficiencies in the g-C_3_N_4_ framework also facilitate enhanced light absorption and efficient charge separation. In addition, strong electron-withdrawing groups (i.e., the cyano groups) were introduced into g-C_3_N_4_; hence, photogenerated electrons in the CB gather on the cyano groups, after which they react with O_2_ to produce O_2_^•−^ at these sites, leading to efficient separation and charge transfer at the surface. Huan *et al*. reported that N-defects at secondary nitrogen sites and C-O-C species act as the active sites for the photocatalytic reaction^82^. However, -OH species caused negative effects, even though there were numerous active sites. Therefore, Bt-OA-CN exhibits excellent photocatalytic activity because there are no –OH species in its framework. The developed Bt-OA-CN preparation method introduces only favourable functionalities that positively affect photocatalytic activity. On the other hand, the remaining h^+^s in the VB of Bt-OA-CN engender this material with a much higher oxidizing ability compared to CN, which may directly degrade BPA. Thus, these favourable dual structural modifications exhibit synergism that accelerates the photocatalytic activity of g-C_3_N_4_.

In summary, dual modifications, namely N-deficiencies and O-dopants, were successfully introduced into the g-C_3_N_4_ framework by the addition of KOH and oxalic acid during the synthesis of g-C_3_N_4_ from urea. The obtained Bt-OA-CN photocatalyst exhibits a modulated energy band structure, separated photo-induced charge carriers, and an expanded light-absorption edge (over 700 nm), which are ascribable to the N vacancies and O dopants. In addition to this dual modification, we confirmed the introduction of cyano groups in the Bt-OA-CN photocatalyst, which serve as strong electron-withdrawing groups that efficiently separate and transfer charge carriers to the surface of the photocatalyst. Bt-OA-CN completely decomposed BPA within 150 min with a high pseudo-first-order reaction rate constant of 0.0225 min^−1^, which is 11.4-times greater than that of CN. The superior photocatalytic activity is ascribable to synergism between these favourable dual structural modifications. PL, TRPL, and (photo)electrochemical techniques reveal the induction of the efficient separation and transfer of charge carriers, while quenching experiments show that both h^+^ and O_2_^•−^ are the predominant oxidizing species in the Bt-OA-CN photocatalyst system. This research provides a simple and easy method for creating structural defects in g-C_3_N_4_, and introduces Bt-OA-CN as a promising highly efficient visible-light-responsive photocatalyst for use in environmental-remediation applications.

## Methods

### Photocatalyst preparation

All reagents used in these experiments were of analytical grade and were used without further purification. Urea, KOH, and oxalic acid were obtained from FUJIFILM Wako Pure Chemicals. CN was prepared by directly heating urea to 550 °C in a muffle furnace for 5 h in a covered alumina crucible at a rate of 3 °C/min. Bt-OA-CN was synthesized as follows: 15 g of urea was dissolved in 30 mL of an aqueous solution of KOH (0.1 g). After stirring for 3 h, the resulting solution was evaporated to dryness at 80 °C using a hot stirrer. The obtained solid was then ground with 5 g of oxalic acid in a mortar. The resultant powder was calcined at 550 °C in a muffle furnace for 5 h at a rate of 3 °C/min. Bt-CN and OA-CN were prepared using the same procedure without the addition of oxalic acid and KOH, respectively.

### Characterization

XRD patterns were recorded on a RIGAKU Ultima IV diffractometer equipped with a Cu Kα radiation source. The morphologies of the samples were examined with a Hitachi S-4000 SEM and a JEOL JEM-1011 TEM. FTIR spectra of the samples were acquired on a SPECTRUM 100 FTIR spectrometer (Perkin Elmer) equipped with an ATR assembly. UV–vis DRS was performed with a JASCO V-750 UV–vis spectrometer equipped with an integrating sphere attachment. XPS was performed using a PHI Quantera SXM photoelectron spectrometer with Al Kα radiation. N_2_ adsorption–desorption isotherms were acquired using a BELSORP-miniII (MicrotracBEL) apparatus. The samples were degassed at 180 °C for 18 h under vacuum prior to any experiment. Specific surface areas and pore-size distributions of the samples were calculated using the BET and BJH methods. PL spectra were acquired on a Shimadzu RF-5300PC system. TRPL experiments were performed on a Quantaurus-Tau C11367 spectrophotometer (Hamamatsu Photonics) at an excitation wavelength of 372 nm. Photoelectrochemical experiments were carried out in a conventional three-electrode system with an electrochemical workstation (VersaSTAT 3, Princeton Applied Research). A saturated Ag/AgCl electrode and a platinum electrode were used as the reference and counter electrodes, respectively. The working electrodes were prepared by depositing the photocatalyst slurry onto ITO as the substrate. An aqueous solution of 0.5 mol/L Na_2_SO_4_ was used as the electrolyte. The working electrode was irradiated by a 300-W Xe lamp (MAX-303, Asahi Spectra) with a UV cut-off filter (L-42, HOYA).

### Evaluating photocatalytic activity

The photocatalytic activities of the synthesized catalysts were evaluated by degrading BPA under visible-light irradiation. In typical run, 30 mg of the photocatalyst was added to 30 mL of BPA solution (10 mg/L) in a 50-mL Pyrex glass reactor. The mixture solution was stirred in dark for 30 min to reach adsorption–desorption equilibrium, after which the sample solution was irradiated using a 300-W Xe lamp (MAX-303, Asahi Spectra) with a UV cut-off filter (L-42, HOYA) while stirring was maintained. After the desired irradiation time, the suspension was centrifuged to remove the photocatalyst and the supernatant was used to determine the concentration of residual BPA by HPLC (GL Science) using an ODS-100V column (150 mm × 4.6 mm i.d., TOSOH) and a GL-7450 UV detector (GL Science) operating at 276 nm. A 6:4 (v/v) mixture of acetonitrile and water was used as the mobile phase at a flow rate of 1.0 mL/min. BPA-mineralization progress was confirmed by measuring the TOC, which was determined with a Shimadzu TOC analyser (TOC–V_E_). For radical trapping experiments, the scavenger for each reactive species was added to the reaction solution in a manner similar to that described for the photocatalytic experiment (above). The scavenger concentrations were: 0.1, 1 × 10^−3^, and 1 × 10^−4^ mol L^−1^ for TBA, AO, and BQ, respectively.

## Supplementary information

Supplementary Information
