# Supplementary material for: Dual-defect-modified graphitic carbon nitride with boosted photocatalytic activity under visible light
Source: Sci Rep. 2019 Oct 16;9:14873. doi: 10.1038/s41598-019-49949-6 (PMC6795803; doi:10.1038/s41598-019-49949-6)
Supplement: Supplementary file 1 — Supplementary Information [file 41598_2019_49949_MOESM1_ESM.pdf]

Supplementary Information

Dual-defect-modified graphitic carbon nitride with boosted photocatalytic activity under visible light

Hideyuki Katsumata[1,*], Fumiya Higashi[1], Yuya Kobayashi[1], Ikki Tateishi[2], Mai Furukawa[1], and Satoshi Kaneco[1,2]

[1]Department of Chemistry for Materials, Graduate School of Engineering, Mie University, Tsu, Mie 514-8507, Japan

[2]Mie Global Environment Center for Education & Research, Mie University, Tsu, Mie 514-8507, Japan

*Corresponding author. hidek@chem.mie-u.ac.jp (H.K.)

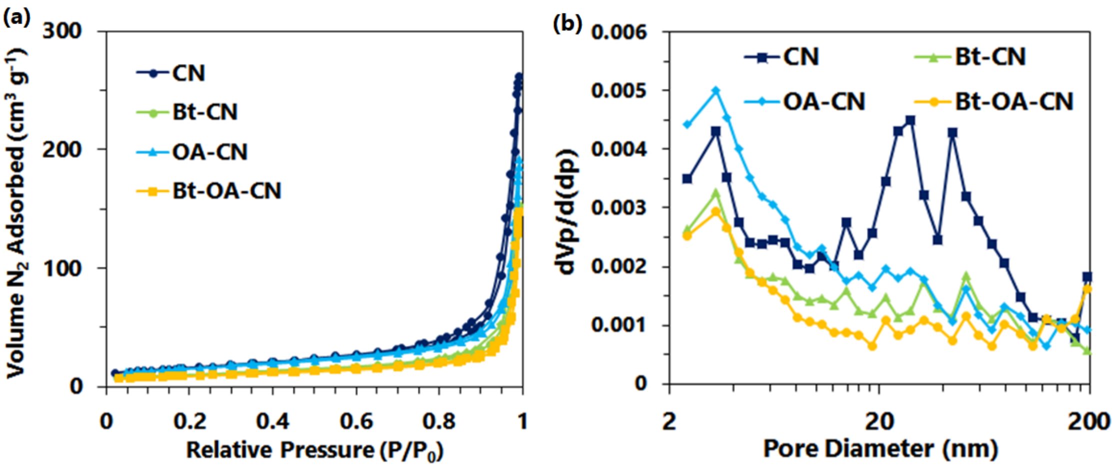

Figure S1. (a) N_2 adsorption-desorption isotherms and (b) BJH pore size distribution curves of CN, Bt-CN, OA-CN and Bt-OA-CN.

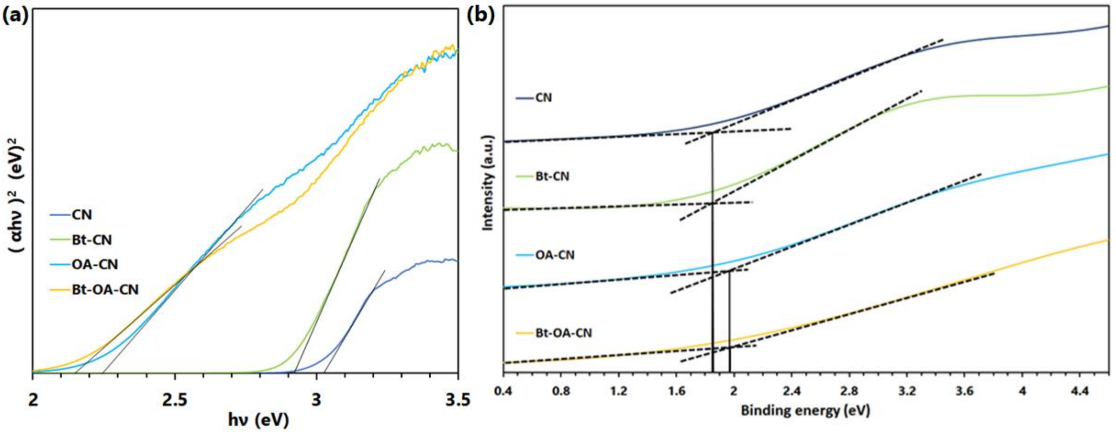

Figure S2. (a) Kubelka-Munk function versus photon energy and (b) VB-XPS spectra of CN, Bt-CN, OA-CN and Bt-OA-CN.

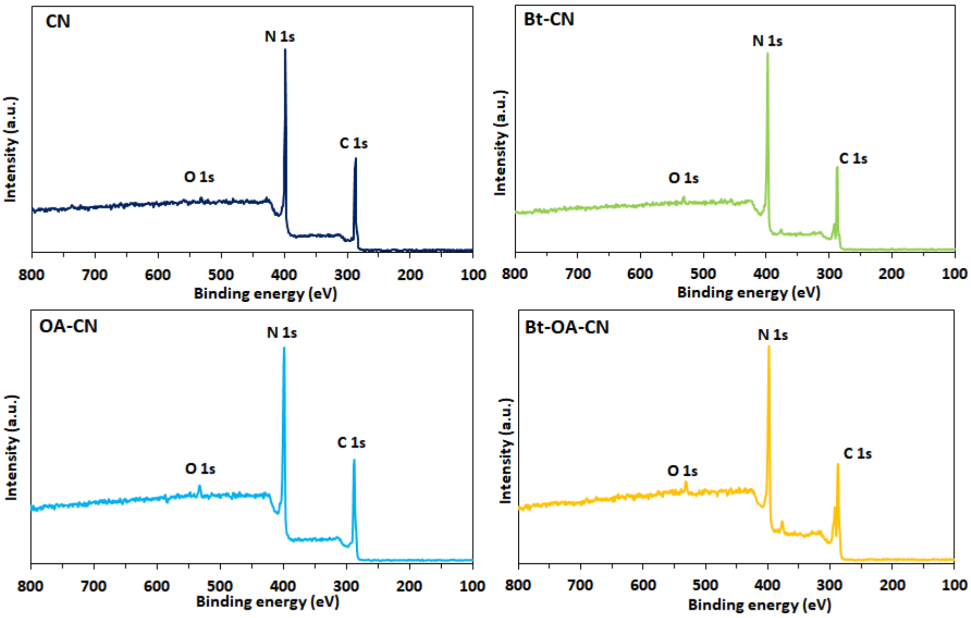

Figure S3. XPS survey spectra of CN, Bt-CN, OA-CN and Bt-OA-CN.

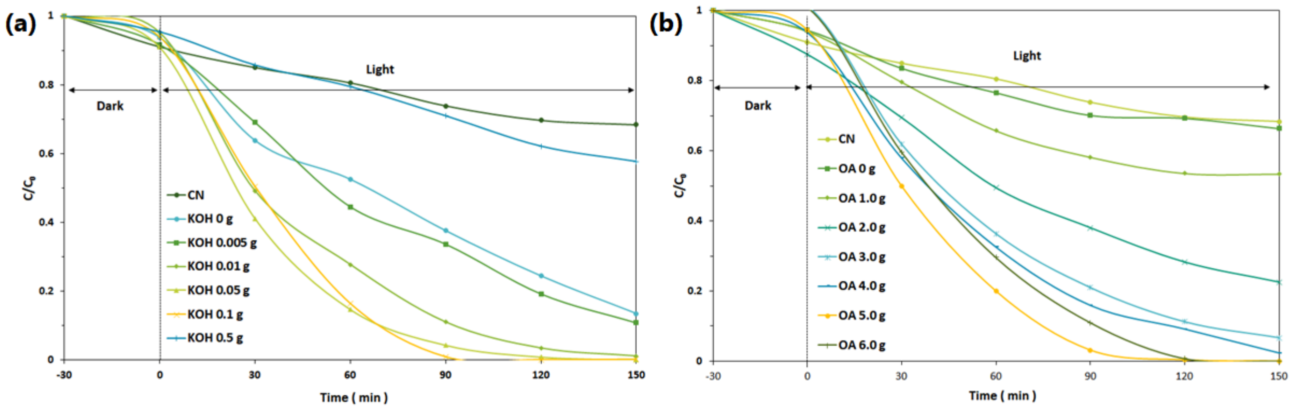

Figure S4. Effect of (a) KOH (OA = 5.0 g) and (b) OA (KOH = 0.1 g) amounts during the thermal polymerization of g-C_3N_4 on the photocatalytic degradation of BPA.

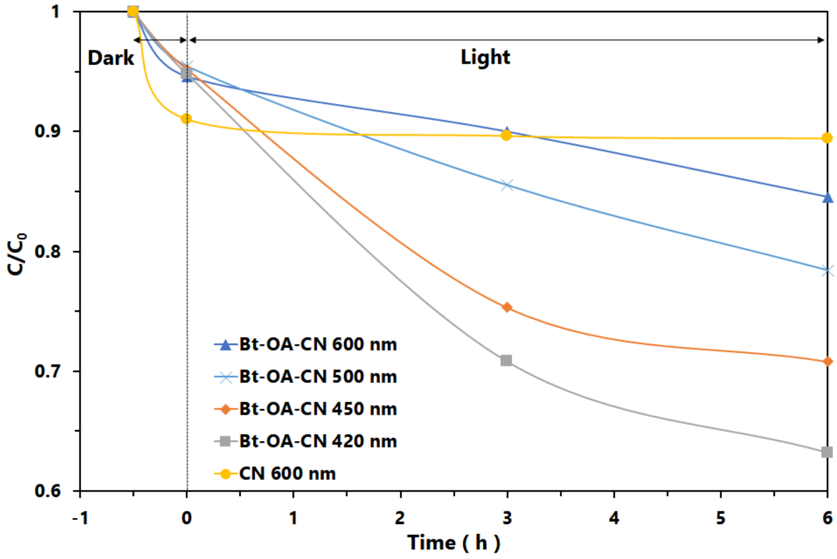

Figure S5. Wavelength dependence of the photocatalytic activity of BPA degradation over Bt-OA-CN and CN.

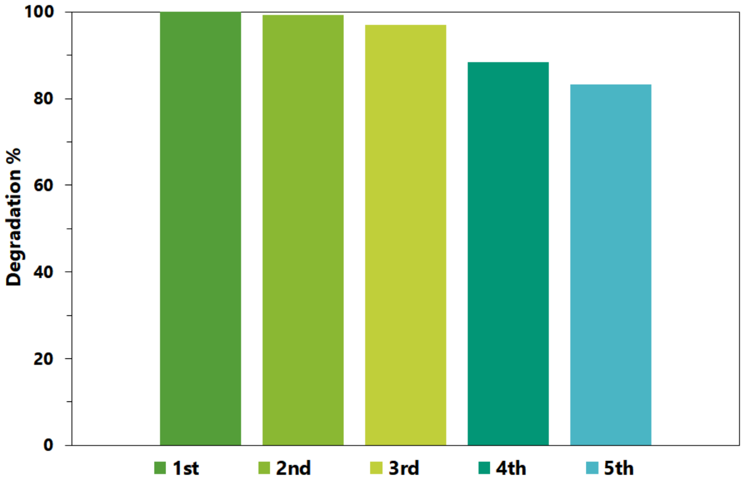

Figure S6. Stability of Bt-OA-CN under visible light. Irradiation time was 150 min for each runs.

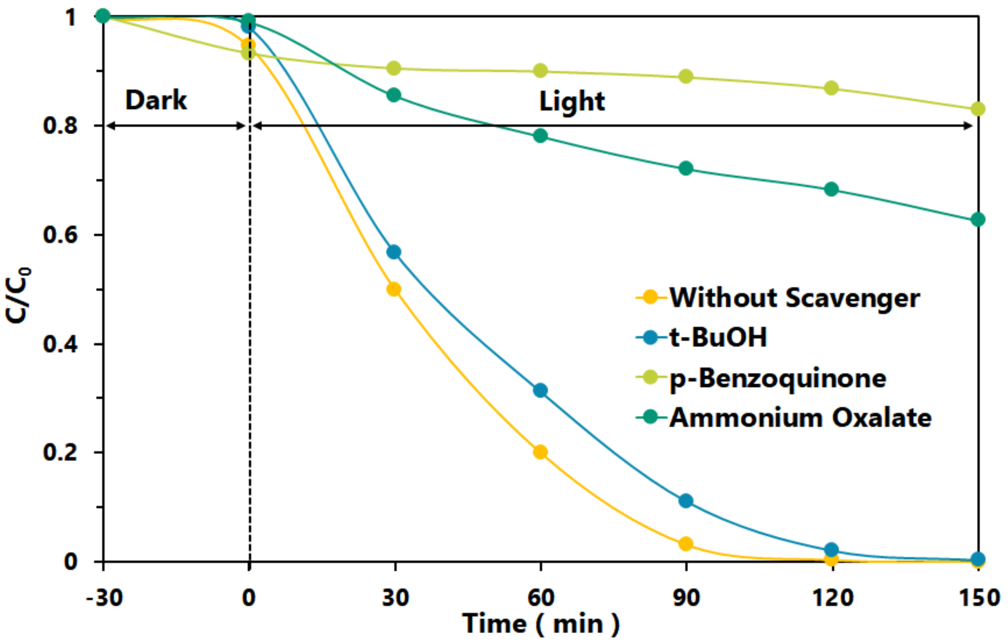

Figure S7. Photocatalytic activity of Bt-OA-CN on the degradation of BPA in the presence of different scavengers under visible light irradiation.

Table S1. Relative ratios of different C species in CN, Bt-CN, OA-CN and Bt-OA-CN based their C1s XPS narrow spectra

Photocatalyst	C=C	N=C-N	C-O-C
CN	0.17	0.83	0
Bt-CN	0.17	0.83	0
OA-CN	0.18	0.77	0.05
Bt-OA-CN	0.17	0.76	0.07

Table S2. Relative ratios and binding energies (B.E.) of different N species in CN, Bt-CN, OA-CN and Bt-OA-CN based their N1s XPS narrow spectra

Photocatalyst	C=N-C (B.E.)	N-$(C)_3$ (B.E.)	N-H (B.E.)	π-excitation (B.E.)
CN	0.77 (398.5)	0.17 (400.3)	0.03 (401.3)	0.03 (404.0)
Bt-CN	0.69 (398.5)	0.20 (399.8)	0.07 (401.1)	0.03 (403.9)
OA-CN	0.67 (398.4)	0.27 (400.1)	0.04 (401.5)	0.03 (404.2)
Bt-OA-CN	0.66 (398.4)	0.24 (399.8)	0.07 (401.2)	0.03 (403.8)

Table S3. Fitted parameters obtained from TRPL spectra of CN, Bt-CN, OA-CN and Bt-OA-CN

Photocatalyst	λ (nm)	<τ> (ns)	τ_1 (ns) (Rel. %)	τ_2 (ns) (Rel. %)	τ_3 (ns) (Rel. %)
CN	462	7.85	0.91 (23.92)	3.90 (45.78)	19.30 (30.30)
Bt-CN	458	3.14	0.44 (39.44)	2.05 (42.60)	11.62 (17.96)
OA-CN	594	0.39	0.11 (71.38)	0.57 (19.92)	2.21 (8.70)
Bt-OA-CN	612	0.21	0.07 (76.13)	0.36 (18.49)	1.65 (5.38)
